# Patterns of Cervical Metastases and Implications for Neck Management in Minor Salivary Gland Cancer Treatment

**DOI:** 10.1002/hed.70044

**Published:** 2025-10-08

**Authors:** Eduardo Wanderley Estanislau da Costa, Pedro Nicolau‐Neto, Paula Moretz‐Sohn, Bernardo Cacciari Peryassu, Emilson de Queiroz Freitas, Luis Felipe Ribeiro Pinto, Fernando Luiz Dias

**Affiliations:** ^1^ Department of Head and Neck Surgery at the Brazilian Air Force Central Hospital Rio de Janeiro Brazil; ^2^ Molecular Carcinogenesis Program Instituto Nacional de Câncer Rio de Janeiro Brazil; ^3^ Marcilio Dias Navy Hospital Rio de Janeiro Brazil; ^4^ Head and Neck Surgery Service Instituto Nacional de Câncer Rio de Janeiro Brazil; ^5^ Department of Biochemistry Rio de Janeiro State University Rio de Janeiro Brazil

**Keywords:** head and neck cancer, lymph node metastasis, minor salivary gland, neck dissection, salivary gland neoplasms

## Abstract

**Objectives:**

Minor salivary gland cancer (MiSGC) is a rare and heterogeneous disease, with different degrees of tumor aggressiveness. The scarce data in the literature show that the presence of lymph node metastases (LNMs) negatively impacts the prognosis. Furthermore, there is a lack of information about the patterns and extension of LNMs in MiSGC. Therefore, it is necessary to identify the clinical and pathological features associated with LNMs in MiSGC to guide the cervical dissection intent of those patients. This study's endpoints were the identification of clinicopathological features associated with LNMs, identification of spread patterns of clinical and occult LNMs, and the impact of LNMs on the patient's outcome.

**Materials and Methods:**

This single‐center retrospective cohort study analyzed data from 437 patients with MiSGC of the oral cavity and oropharynx treated at the Instituto Nacional de Câncer (INCA), the Brazilian National Cancer Institute.

**Results:**

LNMs were observed in 59 patients (13.5%). Cervical levels I and II were the most affected neck levels in the total sample, and in MiSGC of the oral cavity, whereas Levels II and III were the most affected in MiSGC of the oropharynx. Adenocarcinoma histotype, floor of the mouth and base of the tongue subsites, late‐stage tumor, and lymphovascular invasion were associated with LNMs. LNMs were associated with worse overall and disease‐specific survival rates.

**Conclusion:**

There are LNMs spread specific to the site of the MiSGC, with an impact on the patient's prognosis. Adenocarcinoma, tumor subsite, and lymphovascular invasion were associated with LNMs.

## 1 Introduction

Malignant salivary gland cancer (MiSGC) is a heterogeneous group of tumors, accounting for 6% of total head and neck tumors, with approximately 53 000 cases reported annually worldwide [1, 2]. The upper aerodigestive tract has around 400–1000 minor salivary glands, and 90% of them are found in the oral cavity or oropharynx. About 25% of salivary gland tumors are in these minor glands, and 80% are malignant [3].

Cervical lymph node metastases (LNMs) are associated with a worse prognosis for malignant salivary gland tumor patients, and it is estimated that 12%–48% of patients with salivary gland cancer have cervical LNMs [4]. Submandibular and sublingual gland tumors have higher metastatic spread, while the MiSGC have lower rates of cervical LNMs [5]. There is a consensus on the surgical treatment of clinically positive cervical metastases (cN+) in MiSGC. However, the treatment of clinically negative necks (cN0) is controversial. The identification of the predictive factors related to cervical metastases needs to be clarified in the literature. In addition, there is a lack of information regarding the neck dissection extent of patients with MiSGC of the oral cavity or oropharynx. Although some authors and guidelines suggest neck dissection treatment for all cases, others recommend no neck treatment due to the low frequency of metastases [6, 7]. Most authors rely on the biological behavior of salivary gland tumors, especially of MiSGC, to identify the predictive factors related to treatment failures and the impact on survival, thus seeking appropriate treatment intensity [8].

Therefore, in this large single‐center cohort study of MiSGC, we aim to determine the frequency of cervical LNMs, identify spread patterns of clinical and occult LNMs identify the clinical and pathological features associated with cervical LNMs, and assess the features associated with survival rates in MiSGC.

## 2 Materials and Methods

This is a single‐center retrospective study of 457 cases of MiSCG of the oral cavity or oropharynx, treated at the Head and Neck Surgery Service of the Instituto Nacional de Câncer (INCA, Brazil) from January 1995 to December 2015. Patients with synchronous neoplasms were excluded from this study. Therefore, the final sample consisted of 437 male and female different‐aged patients subjected to surgery, radiation therapy, or chemotherapy. The INCA's Department of Pathology evaluated the histopathological profile and tumor histological grade. The study was approved by the hospital's Research Ethics Committee.

The study's primary endpoint was the characterization of clinical and pathological features associated with cervical metastases (pN+), identified by the pathologic analysis of neck dissection nodal specimens. Secondary endpoints were the identification of patients with occult and distant metastases, overall survival (OS), disease‐specific survival (DSS), and disease‐free survival (DFS) rates. The extent of MiSGC was classified according to the 8th edition of the American Joint Committee on Cancer (AJCC) for squamous cell carcinoma of the oral cavity and oropharynx.

The clinicopathological data for patients were obtained from their medical records. The analysis included dependent variables such as cervical LNMs, distant metastases, and mortality. Additionally, independent demographic variables, including sex and age, as well as smoking and drinking histories, were assessed alongside clinical, pathological and treatment variables. Furthermore, surgical margin data were evaluated through perioperative frozen sections, as pathologists are involved in surgical teams for this purpose during all procedures conducted at the institution. This information was also recorded in the patients' medical records.

Descriptive statistical analyses were performed to characterize patients, using frequency distribution and calculation of statistics for quantitative variables. Univariate analyses were performed using the chi‐square test to calculate the odds ratio (OR) and 95% confidence interval (95% CI). Multivariate analysis using the logistic regression model with the backward Wald method was conducted to identify independent factors associated with cervical metastases.

The Kaplan–Meier method and log‐rank test were applied in survival analyses. Patient death was considered an event in the OS analysis. Thus, the patient's follow‐up began immediately after diagnosis and ended on the date of death or the last registration date in the medical record. In the DSS analysis, patient death from MiSCG was considered an event. In DFS, tumor relapse was considered an event. All data were analyzed using IBM‐SPSS version 22, and the significance level was set at 5%.

## 3 Results

Table 1 presents the characteristics of the 437 patients diagnosed with MiSGC included in this study. The mean age of the patients at diagnosis was 53 years (ranging from 10 to 100 years), most patients (66.1%) were younger than 60 years, and female (60%). Most patients (51.5%) had a history of tobacco smoking, and 28.6% reported drinking. Most MiSGC (81.2%) occurred in the oral cavity, and the hard palate was the most commonly affected subsite (43.9%), followed by the buccal mucosa (11.2%). The three most common histological subtypes were adenoid cystic carcinoma (32.5%), adenocarcinoma (31.4%), and mucoepidermoid carcinoma (30.4%), which together accounted for 94.3% of MiSGC. Regarding tumor grade, 172 (39.4%) were classified as high‐grade. It is important to highlight that among the 137 adenocarcinomas we observed, 102 (74.5%) were low‐grade adenocarcinomas, being 88 low‐grade polymorphic subtype and 14 Not Otherwise Specified. We found that only 2% of the cases included cytological analysis, primarily for tumors located in the more anterior subsites of the oral cavity. Due to the tumor locations, incisional or punch biopsies with histological evaluation were performed in 98% of the cases.

**TABLE 1:** Clinicopathological characterization of MiSGC patients included in this study.

Characteristics	Categories	*n*	%
Sex	Female	262	60.0
	Male	175	40.0
Age at diagnosis (years)	< 60	289	66.1
	≥ 60	148	33.9
Tobacco smoking	No	212	48.5
	Yes	225	51.5
Alcohol drinking	No	291	66.6
	Yes	146	33.4
MISGC site	Oral cavity (OC)	355	81.2
	Oropharynx (OP)	82	18.8
MISGC subsite	Hard palate (OC)	192	43.9
	Buccal mucosa (OC)	49	11.2
	Base of the tongue (OP)	37	8.5
	Soft palate (OP)	34	7.8
	Floor of mouth (OC)	33	7.6
	Retromolar trigone (OC)	23	5.3
	Upper alveolar ridge (OC)	22	5.0
	Lip (OC)	17	3.9
	Lower alveolar ridge (OC)	12	2.7
	Palatine tonsil (OP)	11	2.5
	Tongue (OC)	7	1.6
Histological types	Adenoid cystic carcinoma	142	32.5
	Adenocarcinoma	137	31.4
	Mucoepidermoid carcinoma	133	30.4
	Other types	25	5.7
	Carcinoma ex pleomorphic adenoma	7	1.6
	Salivary duct carcinoma	6	1.4
	Clear cell carcinoma	4	0.9
	Epithelial‐myoepithelial carcinoma	4	0.9
	Basal cell adenocarcinoma	2	0.5
	Acinic cell carcinoma	2	0.5
Tumor grade	Low grade	189	43.2
	Intermediate grade	76	17.4
	High grade	172	39.4
T Stage (cTNM)	T1 or T2	185	42.3
	T3 or T4	252	57.7
N Stage (cTNM)	cN0	378	86.5
	cN+	59	13.5
TNM Stage (cTNM)	I or II	169	38.7
	III or IV	268	61.3
T stage (pTNM)^a^	pT1 or pT2	180	46.75
	pT3 or pT4	205	53.25
TNM stage (pTNM)^a^	I or II	163	42.33
	III or IV	222	57.67
Surgical margin status^a^	Negative/close	334	86.7
	Positive	48	12.5
	No information	3	0.8
Bone involvement	No	339	77.6
	Yes	98	22.4
Perineural invasion	No	339	77.6
	Yes	98	22.4
Lymphovascular invasion	No	401	91.8
	Yes	36	8.2
Distant metastasis	No	399	91.3
	Yes	38	8.7

Abbreviations: OC, oral cavity; OP, oropharynx.

^a^ Data based in 385 patients who underwent surgical treatment.

276 (63.15%) patients were categorized as cT1 or cT2, 378 (86.5%) were cN0, and 244 (55.8%) presented clinical early‐stage (I–II). Pathologically, most tumors were classified as pT3 or pT4 (252, 57.7%), cervical LNMs were observed in 59 patients (pN+, 13.5%), and 268 tumors were classified as late‐stage (pTNM III–IV) (61.3%). Tumor‐free surgical margins were observed in 86.7% of the 385 surgically treated individuals; perineural invasion was detected in 22.4%, and lymphovascular invasion was observed in 8.2% of the cases.

**FIGURE 1:**
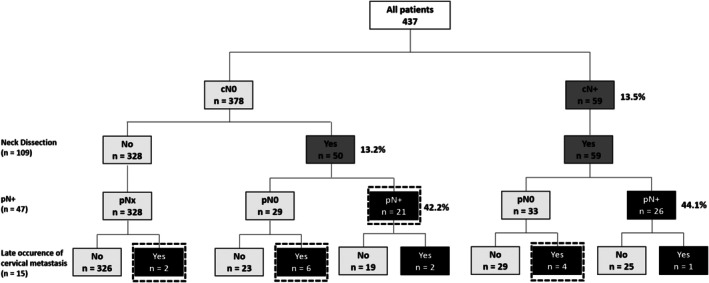
Identification of cervical metastases in MiSGC patients. Schematic representation of the identification of cervical metastases in patients with MiSGC. Patients with cN+ (59 cases) underwent therapeutic treatment of the neck, and among them, 26 had pN+. Regarding cN0 patients, 50 received elective neck treatment, and 21 had pN+. Four cN+/pN0 patients had a late occurrence of cervical metastases, while among the cN0 patients, eight had a late occurrence of cervical metastases. The dashed squares indicates groups of patients classified as having occult metastases.

Among our MiSGC patients, 109 underwent neck dissection. 59 patients with cN+ status received therapeutic neck treatment, and 26 (44%) show cervical LNM (pN+). Additionally, 50 cN0 patients received elective neck treatment. Cervical occult metastases were observed in 33 patients, which included two patients who did not have a neck dissection, 21 cN0 patients, six cN0‐pN0 patients who later had cervical relapse, and four cN+/pN0 patients with cervical relapse (Figure 1). Overall, occult metastases constituted 7.6% of all cases and 55.9% of all cervical LNMs.

Furthermore, 38 patients (8.7%) developed distant metastases, with the lungs being the most affected organ (33 cases, or 86.7%). Among these patients with distant metastasis, nine (2.1%) had also cervical LNM, with no significant association observed between cervical LNM and distant metastases (*p* = 0.055). Oropharyngeal tumors showed a higher frequency of LNMs (21.7% of cases) compared with oral cavity tumors (11.5%) (*p*‐value = 0.013; OR = 2.2).

**FIGURE 2:**
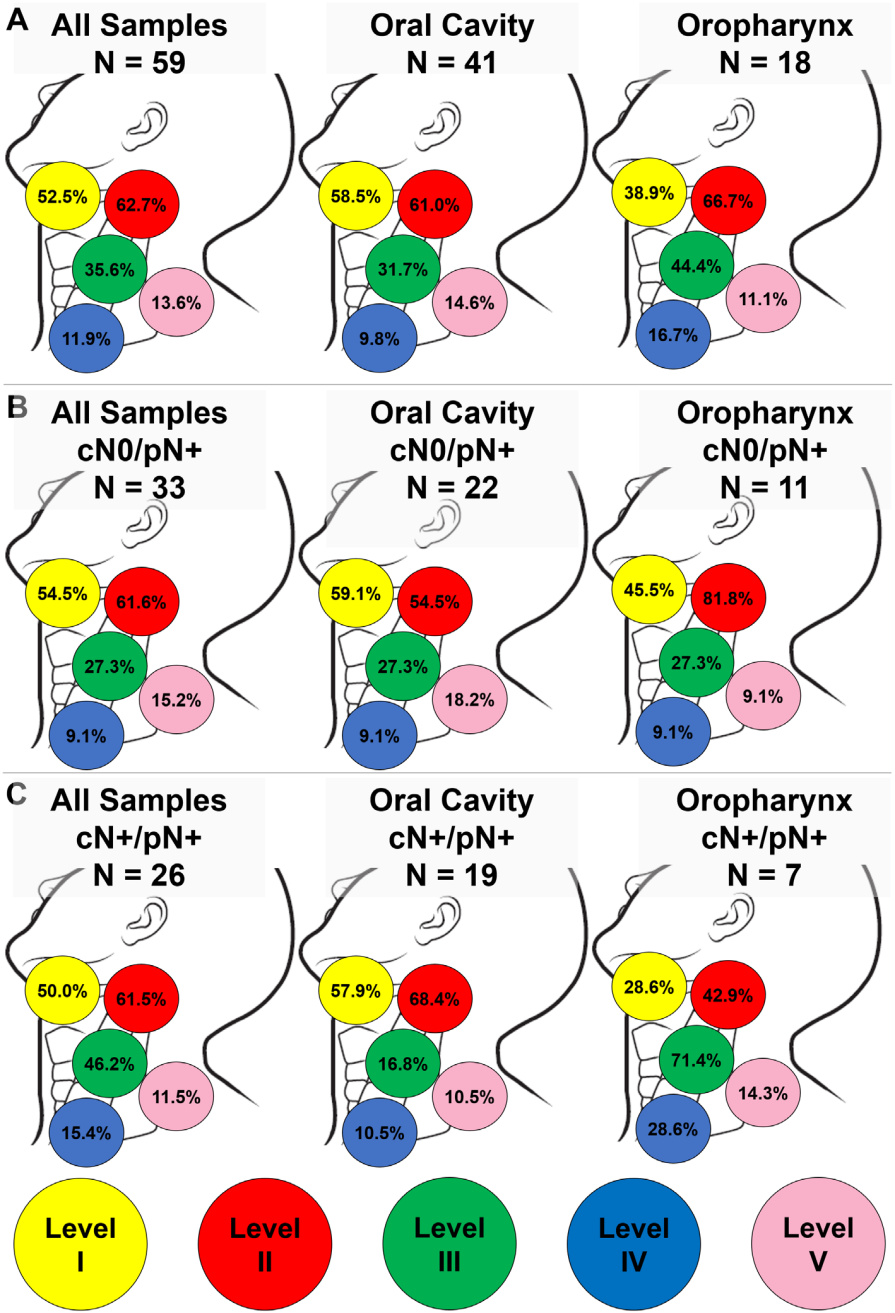
Pattern of cervical metastatic spread at the lymph node levels of the neck. (A) Schematic representation of the neck levels with lymph node metastases according to the MiSGC site. (B) Levels of the neck with lymph node metastases in all cases of occult metastases (cN0/pN+), and according to the MiSGC site. (C) Levels of the neck with lymph node metastases in all patients with metastases diagnosed on clinical examination (cN+/pN+), and according to the MiSGC site. [Color figure can be viewed at wileyonlinelibrary.com]

Analyzing all patients, cervical LNM spread predominantly affected neck levels II (62.7%), I (52.5%), and III (35.6%), and this spread varied according to the MiSGC site. Specifically, neck level I was involved in 58.5% of MiSGC in the oral cavity, and 38.9% of MiSGC cases in the oropharynx (Figure 2A). By comparing the cervical metastatic dissemination between cN+ and occult metastasis patients, we observed a higher frequency of involvement of Levels I, II, and V in samples with occult metastases. In the cases of occult metastases in the oral cavity, Level I was the most commonly involved (59.1%), followed by Levels II (54.5%) and III (27.3%). Among the cases of occult metastases in the oropharynx, involvement of level II was highly frequent (81.8%), followed by Levels I (45.5%) and III (27.3%) (Figure 2B). In the group of patients with clinical metastases (cN+/pN+), Levels II (61.5%), Level I (50.0%), and Level III (46.2%) were the most frequently involved. Among patients with clinical metastases in the oral cavity, Level II was the most commonly involved (68.4%), followed by Levels I (57.9%) and III (36.8%). In the oropharynx group, clinical metastases occurred most frequently at Level III (71.4%), followed by Levels II (42.9%) and I (28.6%) (Figure 2C).

Aiming to describe the clinical and pathologic features associated with cervical LNMs, we observed in the univariate analyses that male sex (OR = 2.1; *p* = 0.007), alcohol drinking history (OR = 3.0; *p* < 0.001), adenocarcinoma histotype (OR = 2.6; *p* < 0.001), adenoid cystic carcinoma histotype (OR 0.4; *p* = 0.006), MiSGC of the oropharynx (OR = 2.2; *p* = 0.013), MiSGC of the floor of the mouth (OR = 4.3; *p* < 0.001), MiSGC of the base of the tongue (OR = 2.1; *p* = 0.012), cT3 or cT4 (OR = 2.2; *p* = 0.004), cN+ (OR = 12.4; *p* < 0.001), advanced clinical tumor stage (cTNM) (III or IV) (OR = 5.4; *p* < 0.001), pT3 or pT4 (OR = 2.7; *p* = 0.002), advanced pathological tumor stage (pTNM) (III or IV) (OR = 10.7; *p* = 0.011), and lymphovascular involvement (OR = 4.4; *p* < 0.001) were associated with cervical LNM. In the multivariate analysis, the independent factors associated with cervical LNMs were adenocarcinoma histotype (OR = 2.9; *p* = 0.002), MiSGC of the floor of the mouth (OR = 4.0; *p* = 0.007), MiSGC of the base of the tongue (OR = 2.6; *p* = 0.045), cN+ (OR = 8.3; *p* < 0.0001), advanced pathological tumor stage (III or IV) (OR = 5.0; *p* = 0.004), and lymphovascular involvement (OR = 2.9; *p* < 0.020) (Table 2).

**TABLE 2:** Univariate and multivariate logistic regression analyses of association between clinicopathological features and cervical LNMs.

Characteristics	Risk factor	Cervical LNM cases	Univariate			Multivariate		
			*p*	OR	95% CI	*p*	OR	95% CI
Sex	Male	33	**0.007**	2.1	1.2–3.7			
Age at diagnosis (years)	≥ 60	16	0.239	0.7	0.4–1.3			
Tobacco smoking	Yes	34	0.310	1.3	0.8–2.3			
Alcohol drinking	Yes	33	**< 0.001**	3.0	1.7–5.2			
MISGC site	Oropharynx (OP)	18	**0.013**	2.2	1.2–4.0			
MISGC subsite	Hard palate (OC)	12	**< 0.001**	0.3	0.1–0.5			
	Base of the tongue (OP)	10	**0.012**	2.1	2–5.8	**0.045**	2.6	1.02–6.7
	Floor of mouth (OC)	12	**< 0.001**	4.3	2.0–9.4	**0.007**	4.0	1.5–10.9
Histological types	Adenocarcinoma	30	**0.001**	2.6	1.5–4.6	**0.002**	2.9	1.5–5.7
	Adenoid cystic carcinoma	10	**0.006**	0.4	0.2–0.8			
	Mucoepidermoid carcinoma	17	0.989	1.0	0.6–1.8			
Tumor grade	High grade	30	0.052	1.7	0.9–3.0			
T Stage (cTNM)	T3 or T4	32	**0.004**	2.2	1.3–3.9			
N Stage (cTNM)	cN+	30	**< 0.001**	12.4	6.6–23.5	**< 0.001**	8.3	4.1–17.1
TNM stage (cTNM)	III or IV	46	**< 0.001**	5.4	2.8–10.3			
T stage (pTNM)	T3 or T4	45	**0.002**	2.7	1.5–5.0			
TNM stage (pTNM)	III or IV	55	**< 0.001**	10.7	3.8–30.0	**0.004**	5.0	1.7–14.9
Distant metastasis	Yes	9	0.055	2.16	0.94–4.87			
Margin status	Positive	18	0.645	1.2	0.6–2.1			
Bone involvement	Yes	15	0.553	1.2	0.6–2.3			
Perineural invasion	Yes	18	0.109	1.6	0.9–3.0			
Lymphovascular invasion	Yes	13	**< 0.001**	4.4	2.1–9.2	**0.020**	2.9	1.2–7.3

*Note*: Bold values: *p*‐value < 0.05 was considered significant.

Abbreviations: 95% CI, 95% confidence interval; OR, odds ratio. bold values: *p*‐value < 0.05 was considered significant.

We observed the impact of cervical LNMs on the survival rates of MiSGC patients. Our findings revealed that cervical LNMs resulted in a mean OS of 11.3 years for pN+ patients, while those with no cervical metastases showed a mean survival of 15.9 years, with HR = 2.1 (*p*‐value < 0.001). In DSS analysis, patients with cervical LNMs had a mean DSS of 13.8 years for pN+ patients, while those without cervical metastases had a mean DSS of 19.5 years (HR = 1.8; *p*‐value = 0.015). Similar data was observed in DFS analysis, when pN+ patients present worse survival rates than pN0 patients (HR = 2.2; *p* = 0.038).

**FIGURE 3:**
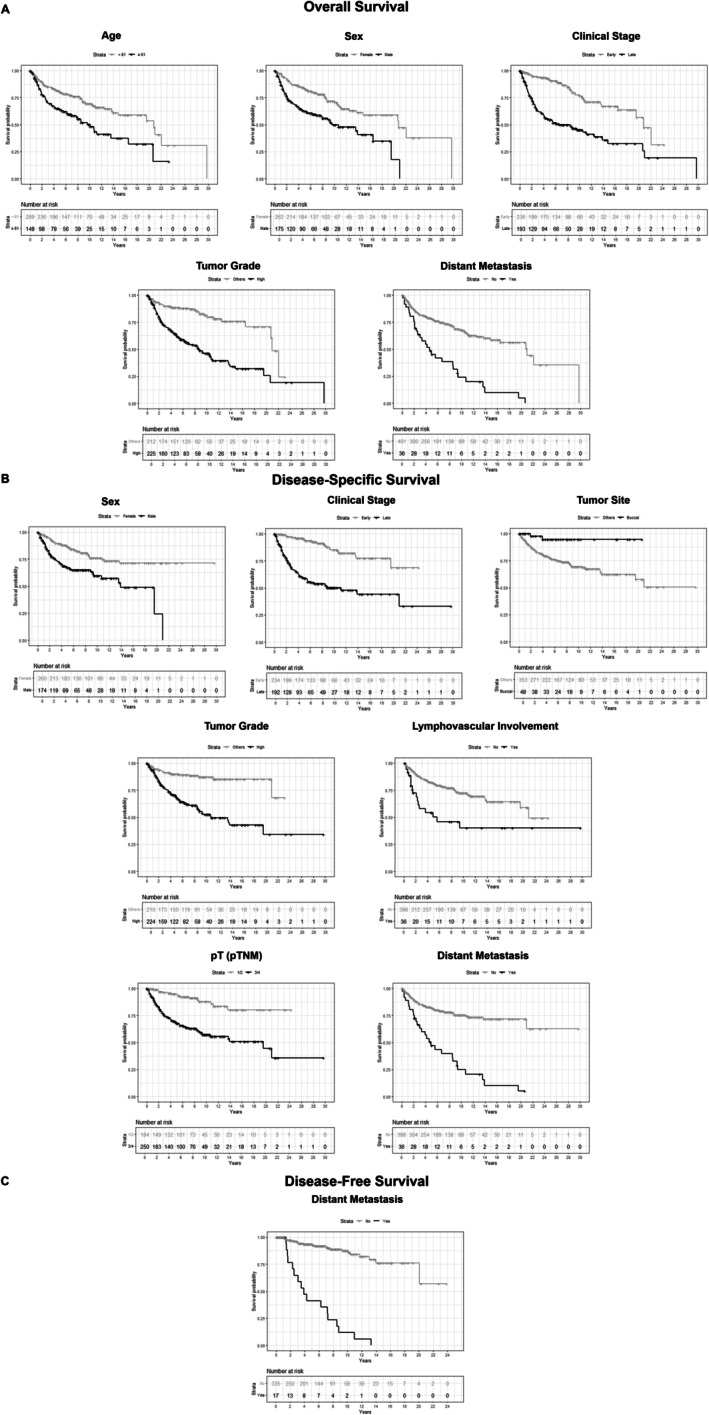
Kaplan–Meier curves showing the impact of metastases on the prognosis of patients with MiSGC. (A) Overall survival analysis pointed out patients older than 61 years (HR = 1.6), male sex (HR = 1.8), advanced clinical stage (HR = 2.9), high‐grade tumor (HR = 2.5), and distant metastasis (HR = 1.7) as independent prognostic markers. (B) Disease‐specific survival analysis pointed‐out male sex (HR = 1.9), advanced clinical stage (HR = 4.9), tumor located in the buccal mucosa (HR = 0.1), high‐grade tumor (HR = 2.8), lymphovascular involvement (HR = 2.0), pT 3/4 (HR = 5.1) and distant metastasis (HR = 2.4) as independent prognostic markers. (C) Distant metastasis (HR = 9.5) was classified as an independent prognostic marker of disease‐free survival.

Multivariate survival analyses show us that male sex (OS—HR = 1.8; *p* < 0.001; DSS—HR = 1.9; *p* = 0.002), patients older than 60 years (OS—HR = 1.6; *p* = 0.005), buccal mucosa site (DSS—HR = 0.1; *p* = 0.015), late clinical stage (OS—HR = 2.9; *p* < 0.001; DSS—HR = 4.9; *p* < 0.001), pT 3/4 (DSS—HR = 2.1; *p* < 0.001), high‐grade tumor (OS—HR = 2.5; *p* < 0.001; DSS—HR = 2.8; *p* < 0.001), lymphovascular involvement (DSS—HR = 2.0; *p* = 0.018) and distant metastasis (OS—HR = 1.7; *p* < 0.001; DSS—HR = 2.4; *p* < 0.001; DFS—HR = 9.5; *p* < 0.001) were independently associated with survival rates in our cohort of MiSGC patients (Table 3; Figure 3).

**TABLE 3:** Cox's regression model analyses to overall survival (OS), disease‐specific survival (DSS) and disease‐free survival (DFS).

Characteristics	Risk factor	Overall survival				Disease‐specific survival				Disease‐free survival			
		Univariate		Multivariate		Univariate		Multivariate		Univariate		Multivariate	
		HR (95% CI)	*p*	HR (95% CI)	*p*	HR (95% CI)	*p*	HR (95% CI)	*p*	HR (95% CI)	*p*	HR (95% CI)	*p*
Sex	Female	Ref	***p* < 0.001**	Ref	***p* < 0.001**	Ref	***p* < 0.001**	Ref	***p* = 0.002**	Ref	*p* = 0.56		
	Male	**2.2 (1.60–3.13)**		**1.8 (1.30–2.57)**		**2.4 (1.61–3.49)**		**1.9 (1.26–2.94)**		1.2 (0.68–2.24)			
Age	≤ 60	Ref	***p* < 0.001**	Ref	***p* = 0.005**	Ref	***p* = 0.006**			Ref	*p* = 0.78		
	> 60	**2.0 (1.44–2.80)**		**1.6 (1.15–2.26)**		**1.7 (1.17–2.55)**				0.9 (0.48–1.78)			
Alcohol drinking	No	Ref	***p* = 0.036**			Ref	*p* = 0.09			Ref	*p* = 0.07		
	Yes	**1.4 (1.02–2.00)**				1.4 (0.95–2.06)				0.8 (0.41–1.47)			
Tobbaco smoking	No	Ref	***p* = 0.005**			Ref	***p* = 0.011**			Ref	*p* = 0.08		
	Yes	**1.6 (1.15–2.26)**				**1.7 (1.12–2.45)**				0.6 (0.34–1.11)			
Tumor site	Others	Ref				Ref		Ref		Ref			
	Buccal mucosa	0.5 (0.25–1.06)	***p* = 0.05**			**0.2 (0.04–0.66)**	***p* = 0.002**	**0.1 (0.04–0.71)**	***p* = 0.015**	0.7 (0.26–2.09)	*p* = 0.58		
	Floor of mouth	**1.9 (1.11–3.17)**	***p* = 0.01**			**2.0 (1.15–3.56)**	***p* < 0.001**			**2.7 (1.21–6.17)**	***p* = 0.009**		
Histological subtypes	ADNC	Ref				Ref				Ref			
	CAC	1.4 (0.98–2.10)	*p* = 0.27			**1.7 (1.11–2.68)**	***p* = 0.024**			1.7 (0.88–3.14)	*p* = 0.06		
	CME	**0.5 (0.27–0.77)**	***p* = 0.002**			0.4 (0.22–0.79)	*p* = 0.35			0.3 (0.12–0.80)	***p* = 0.030**		
	Others	1.4 (0.75–2.69)	*p* = 0.97			1.2 (0.52–2.70)	*p* = 0.67			1.0 (0.24–4.51)	*p* = 0.84		
cT	1/2	Ref	***p* < 0.001**			Ref	***p* < 0.001**			Ref	*p* = 0.65		
	3/4	**3.0 (2.12–4.16)**				**3.7 (2.46–5.44)**				1.6 (0.85–2.91)			
cN+	No	Ref	***p* < 0.001**			Ref	***p* < 0.001**			Ref	*p* = 0.32		
	Yes	**2.7 (1.78–3.97)**				**3.3 (2.16–5.16)**				1.5 (0.62–3.45)			
cTNM	I/II	Ref	***p* < 0.001**	Ref	***p* < 0.001**	Ref	***p* < 0.001**	Ref	***p* < 0.001**	Ref	*p* = 0.57		
	III/IV	**3.6 (2.53–5.18)**		**2.9 (2.06–4.27)**		**5.3 (3.36–8.27)**		**4.9 (2.91–8.33)**		1.8 (0.99–3.14)			
pT	1/2	Ref	***p* < 0.001**			Ref	***p* < 0.001**	Ref	***p* < 0.001**	Ref	*p* = 0.56		
	3/4	**3.4 (2.24–5.14)**				**4.5 (2.65–7.49)**		**2.1 (1.19–3.67)**		1.8 (0.99–3.28)			
pTNM	I/II	Ref	***p* < 0.001**			Ref	***p* < 0.001**			Ref	***p* = 0.001**		
	III/IV	**4.1 (2.95–6.48)**				**7.0 (3.64–13.4)**				**2.8 (1.47–5.47)**			
High‐grade tumor	No	Ref	***p* < 0.001**	Ref	***p* < 0.001**	Ref	***p* < 0.001**	Ref	***p* < 0.001**	Ref	***p* = 0.035**		
	Yes	**4.1 (2.86–5.79)**		**2.5 (1.68–3.72)**		**4.0 (2.67–5.99)**		**2.8 (1.74–4.79)**		**2.0 (1.13–3.65)**			
Lymphovascular involvement	No	Ref	***p* < 0.001**			Ref	***p* = 0.001**	Ref	***p* = 0.018**	Ref	*p* = 0.16		
	Yes	**2.2 (1.40–3.57)**				**2.6 (1.53–4.34)**		**2.1 (1.14–4.00)**		1.3 (0.69–2.35)			
Treatment	Surgery	Ref				Ref				Ref	*p* = 0.42		
	Surgery + radiotherapy	**1.8 (1.24–2.73)**	***p* < 0.001**			**2.1 (1.27–3.29)**	***p* < 0.001**			1.6 (0.89–12.86)			
	Exclusive radiotherapy	**5.1 (3.69–9.14)**	***p* < 0.001**			**7.8 (4.63–13.1)**	***p* < 0.001**			Not applicable			
Cervical metastasis	No	Ref	***p* < 0.001**			Ref	***p* = 0.015**			Ref	***p* = 0.038**		
	Yes	**2.1 (1.40–3.31)**				**1.8 (1.11–2.88)**				**2.2 (1.014–4.83)**			
Distant metastasis	No	Ref	***p* < 0.001**	Ref	***p* = 0.008**	Ref	***p* < 0.001**	Ref	***p* < 0.001**	Ref	**< 0.001**	Ref	***p* < 0.001**
	Yes	**3.4 (2.26–5.09)**		**1.7 (1.15–2.70)**		**4.4 (2.9–6.7)**		**2.4 (1.45–4.02)**		**12.9 (7.03–23.61)**		**9.5 (4.80–18.91)**	

*Note*: Bold values: *p*‐value < 0.05 was considered significant.

Abbreviations: 95% CI, 95% confidence interval; CI, confidence interval; HR, hazard ratio; Ref, Reference = 1. Bold values: *p*‐value < 0.05 was considered significant.

## 4 Discussion

MiSGC is a heterogeneous group of tumors, with scarce data in the literature [8], and the identification of clinical and pathologic characteristics related to cervical LNM and prognosis is pivotal to improving patient management. This study is one of the largest single‐center works on MiSGC, having assessed 437 patients with MiSGC of the oral cavity (81.2%) and oropharynx (18.8%) treated between 1995 and 2015 at INCA. We identified that 13.5% of MiSGC patients developed cervical LNMs, which was associated with a worse prognosis. Other studies have reported an overall rate of cervical LNMs in MiSGC, ranging from 3.3% to 21.0% [5, 6, 9, 10, 11, 12, 13, 14, 15]. It should be highlighted that, by comparing our data with these rates described in the literature, Hyam et al. [11] found 3.3% of LNMs in a study with 30 patients over a 22‐year follow‐up period, whereas Spiro et al. [15] found 21% on several sites in a cohort of 378 patients in 45 years of follow‐up, which could explain this difference in incidence rates among studies.

We observed the main features associated with cervical metastases in MiSGC: adenocarcinoma histotype, cN+, late‐stage tumor, tumor in the floor of the mouth and base of the tongue subsites, and lymphovascular invasion, with oropharyngeal tumors presenting significantly higher risk of cervical metastases than patients with MiSGC in the oral cavity. Lee et al. [13], in a study with 60 patients, observed an association between cervical metastases and tumor stage. Male gender, histologic type, T classification, and oropharyngeal site were also associated with cervical metastases [12].

We observed that 7.6% of MiSGC cases presented occult metastases, in line with previous studies, which showed between 2.4% [5] and 6.7% [13] of MiSGC patients with this feature. Occult cervical metastasis was already related to undifferentiated carcinoma, squamous cell carcinoma, high‐grade mucoepidermoid carcinoma, adenocarcinoma, carcinoma ex‐pleomorphic adenoma, salivary duct carcinoma, and high‐grade differentiated tumors [4, 16]. In our analysis, we analyzed a higher rate of adenocarcinoma and mucoepidermoid tumors compared to other studies, and we also observed that these histological subtypes were associated with occult metastases.

Although the literature about squamous cell carcinoma of the oropharynx describes a higher frequency of metastatic spread at Levels II, III, and IV [17], we also found different neck levels involvement related to the MiSGC in the oropharynx. Our data suggest the necessity of planning neck dissection in MiSGC of the oropharynx including neck level I, due its significant involvement in our study (38.9%). Still, concerning the patterns of neck metastatic spread, in our total sample, the most affected levels were II, I, and III respectively, and Level II was the most commonly involved in the oral cavity and oropharynx. Note that 55.9% of the cases with cervical LNMs showed only one positive lymph node. After studying 12 MiSGC patients with cervical LNMs, Lopes et al. [18] observed involvement of neck level II in four patients (33.3%), Level III in four cases (33.3%), and Level IV in two patients (16.7%). The level of neck involvement was unknown in one patient.

Selective neck dissection is currently recommended based on the primary tumor sites and their main associated factors, such as size, histological type, and tumor grade [4, 19]. In addition, treatment of the neck is suggested when there is clinical or radiological evidence of regional metastases in MiSGC or when the risk of subclinical disease in a clinically negative neck exceeds 15%–20% [20]. Here, we highlight the recommendation for elective neck dissection in the presence of predictive factors significantly associated with cervical LNMs, in addition to those that had been previously described [21, 22, 23]. For cervical LNMs, modified radical cervical dissection at Levels I, II, III, IV, and V should be recommended according to the international literature [4, 5, 8].

Regarding the patients with occult metastases, Levels II, I, and III, in order of frequency, were again the most frequently involved. Lee et al. [13] observed the involvement of cervical levels in four occult metastases and four cases of regional recurrence. However, they did not specify which cases referred to intraoral and nasosinusal tumors. Among the four cases of occult metastases, two were classified as IIa and III, one as IIa, and one as III. Regarding regional recurrence, one case was classified as IIa, III, Ib, and IIa, and one as Ib and III, indicating that levels IIa and III were the most frequently affected. However, the authors included tumors at more than one site and with different patterns of lymphatic dissemination. It was not possible to compare them with specific data obtained for the oral cavity and oropharynx, even though they described the cervical levels with metastatic spread.

Our data indicate that patients with cervical LNM showed lower mean survival rates in OS, DSS, and DFS, reinforcing the relevance of LNM to patients' prognosis. The studies by Lloyd et al., Copelli et al., and Hay et al. [6, 12, 24] also reported a negative and significant impact of LNM on OS. The studies by Copelli et al. and Hay et al. [6, 24] also reported a negative and significant impact of LNM on DSS. Likewise, Elhusseiny et al. [25] also observed that cervical LNMs reduce DSS, especially after 10 years of follow‐up. Our data revealed a significant impact of distant metastasis on prognosis, resulting in drastically decreased survival rates among our patients. Copelli et al. and Hay et al. [6, 24] did not find an association between distant metastasis and survival; however, Lee et al. [13] concluded that the quality of survival for patients who developed distant metastasis is unsatisfactory compared to those who did not. Nam et al. [26] described that distant metastasis has a significant effect on reducing OS and DSS, as shown in this study. In the study by Brajkovic et al. [14], distant metastasis was cited as the leading cause of death and treatment failure.

In the present study, high‐grade tumors were found in 39.4% of the cases. Previous research has reported that the frequency of high‐grade MiSGC ranges from 22.0% to 70.5% [6, 12, 14], and our findings fall within this range. We also noted an association between high‐grade MiSGC and OS as well as DSS, although no association was found with DFS. Similar results have been reported by Hay et al. and Brajkovic et al. [6, 14], where high‐grade tumors were associated with OS and DSS but not with DFS. In addition, Brajkovic et al. [14] suggested that elective neck dissection should be considered as a therapeutic approach for selected cases of high‐grade MiSGC.

This study has some limitations, such as the retrospective collection of data. Notwithstanding these limitations, this study included a significant number of patients treated at a single institution, showing the importance of cervical LNMs and identifying predictive factors, and also revealed frequent lymph node involvement in cN+ and cN0 patients. Our findings could be useful for the management of the neck in patients with MiSGC of the oral cavity and oropharynx.

## 5 Conclusion

We observed a frequency of 13.5% of LNMs in MiSGC, including occult metastases, which was associated with adenocarcinoma histotype, tumor site, advanced stage, and lymphovascular invasion. We also described a specific spread pattern regarding MiSGC sites, which could be useful for neck dissection planning. Finally, LNMs impacted negatively the OS, DSS, and DFS rates.

## Conflicts of Interest

The authors declare no conflicts of interest.

## Data Availability

The data that support the findings of this study are available on request from the corresponding author. The data are not publicly available due to privacy or ethical restrictions.
